# Fraxinellone Attenuates Rheumatoid Inflammation in Mice

**DOI:** 10.3390/ijms19030829

**Published:** 2018-03-13

**Authors:** Seung Min Jung, Jaeseon Lee, Seung Ye Baek, Juhyun Lee, Se Gwang Jang, Seung-Min Hong, Jin-Sil Park, Mi-La Cho, Sung-Hwan Park, Seung-Ki Kwok

**Affiliations:** 1 Division of Rheumatology, Department of Internal Medicine, Yonsei University College of Medicine, 15-1 Yonseo-ro, Seodaemun-gu, Seoul 03722, Korea; jsmin00@yuhs.ac; 2 Rheumatism Research Center, Catholic Institutes of Medical Science, College of Medicine, The Catholic University of Korea, 222 Banpo-daero, Seocho-gu, Seoul 06591, Korea; llooo@naver.com (J.L.); syeee23@catholic.ac.kr (S.Y.B.); um26sw@hanmail.net (J.L.); yourelite@naver.com (S.G.J.); dkask0809@naver.com (S.-M.H.); wlstlf81@catholic.ac.kr (J.-S.P.); iammila@catholic.ac.kr (M.-L.C.); rapark@catholic.ac.kr (S.-H.P.); 3 Division of Rheumatology, Department of Internal Medicine, Seoul St. Mary’s Hospital, College of Medicine, The Catholic University of Korea, 222 Banpo-daero, Seocho-gu, Seoul 06591, Korea

**Keywords:** fraxinellone, collagen-induced arthritis, rheumatoid arthritis, inflammatory arthritis, osteoclastogenesis

## Abstract

This study aimed to evaluate the therapeutic effect of fraxinellone on inflammatory arthritis and identify the underlying mechanisms. Fraxinellone (7.5 mg/kg) or a vehicle control was injected into mice with collagen-induced arthritis (CIA). The severity of arthritis was evaluated clinically and histologically. The differentiation of CD4^+^ T cells and CD19^+^ B cells was investigated in the presence of fraxinellone. Osteoclastogenesis after fraxinellone treatment was evaluated by staining with tartrate-resistant acid phosphatase (TRAP) and by measuring the mRNA levels of osteoclastogenesis-related genes. Fraxinellone attenuated the clinical and histologic features of inflammatory arthritis in CIA mice. Fraxinellone suppressed the production of interleukin-17 and the expression of *RAR-related orphan receptor γ t* and phospho-signal transducer and activator of transcription 3 in CD4^+^ T cells. CD19^+^ B cells showed lower expression of *activation-induced cytidine deaminase* and *B lymphocyte-induced maturation protein-1* after treatment with fraxinellone. The formation of TRAP-positive cells and the expression of osteoclastogenesis-related markers were reduced in the presence of fraxinellone. Inhibition of interleukin-17 and osteoclastogenesis was also observed in experiments using human peripheral mononuclear cells. Fraxinellone alleviated synovial inflammation and osteoclastogenesis in mice. The therapeutic effect of fraxinellone was associated with the inhibition of cellular differentiation and activation. The data suggests that fraxinellone could be a novel treatment for inflammatory arthritis, including rheumatoid arthritis.

## 1. Introduction

Rheumatoid arthritis (RA) is a systemic autoimmune disease characterized by inflammatory polyarthritis that lead to joint destruction and functional disability. The pathogenesis of RA still remains to be determined, but the interplay between various immune cells would be critical in the development and progression of RA [1]. The pathologic roles of T cells have been extensively studied, and the inflammatory subset of T cells producing interleukin (IL)-17 (Th17 cells) is considered to play a key role in the pathogenesis of inflammatory arthritis [2]. IL-17 stimulates RA synoviocytes to produce IL-6, which promotes the production of proinflammatory mediators, such as IL-1β, tumor necrosis factor-alpha (TNF-α), and matrix metalloproteinase, and accelerates synovial inflammation and bone destruction. IL-6 also induces reciprocal differentiation of CD4^+^ T cells into Th17 cells. IL-17 induces the expression of receptor activator of nuclear factor-κB (RANK) ligand (RANKL), and accelerates erosion of bone. In addition, B cells producing specific autoantibodies contribute to the pathogenesis of RA. Although the pathologic role of B cells is unclear, targeted-therapy-blocking B cells has long-lasting therapeutic effects in patients with RA [3,4]. Treatment of RA aims to suppress the inflammatory response provoked by these immune cells, and thus reduce the synovitis and osteoclastogenesis [5].

Recent advances in RA treatment have generated greater therapeutic opportunity, which has led to improved clinical outcomes in RA patients. Drug therapy in RA aims to reach and maintain a disease remission state via treatment with disease modifying antirheumatic drugs (DMARDs). However, some patients still show inadequate response to DMARDs, or the use of DMARDs is often limited due to comorbidities and drug complications. Thus, there are efforts to investigate novel therapeutic agents to treat RA in an effective and safe way. For development of new drugs, traditional medicine could be a promising approach.

The root bark of *Dictamnus dasycarpus*, a plant widely distributed throughout Korea and China, is a traditional herb used to treat inflammatory conditions. There have been recent efforts to identify the active components of *D. dasycarpus*, and the identified compounds have included dictamdiol, rutevin, limonoid, fraxinellone, fraxinellonone, obacunone, and dictamine [6,7]. Among these constituents, fraxinellone is suggested to have anti-inflammatory and neuroprotective effects [8,9,10,11,12,13,14]. Recent studies have suggested that fraxinellone has a potential therapeutic effect in animal models with inflammatory diseases. Fraxinellone demonstrated therapeutic efficacy in mice with experimental colitis, and T cell-dependent hepatitis [13,15]. In an allergy murine model, treatment with *D. dasycarpus* extract was also effective [16].

This study aimed to evaluate the therapeutic effect of fraxinellone in mice with inflammatory arthritis and identify the underlying mechanisms that contribute to alleviating inflammatory arthritis. We compared clinical arthritis between collagen-induced arthritis (CIA) mice treated with fraxinellone and a vehicle control, and investigated the inhibitory effects of fraxinellone on inflammatory immune cell functions.

## 2. Results

### 2.1. Fraxinellone Alleviates Inflammatory Arthritis in CIA Mice

**Figure 1:**
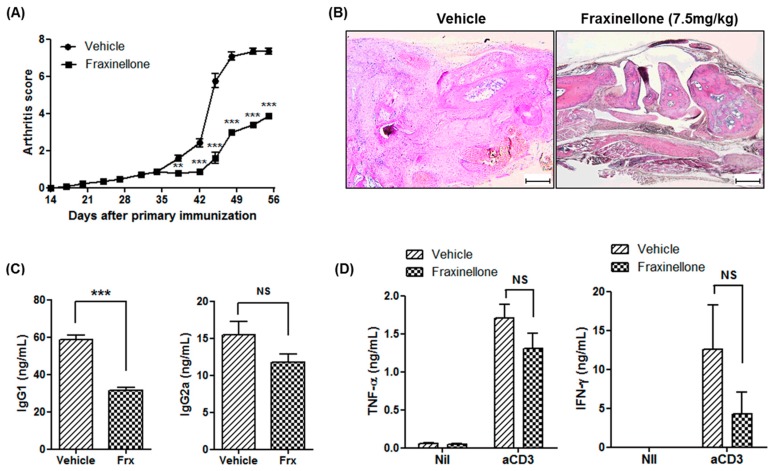
Fraxinellone attenuates inflammatory arthritis in mice. (**A**) Mice with collagen-induced arthritis (CIA) were treated with fraxinellone (7.5 mg/kg) or the vehicle control (*n* = 10 in each group). The arthritic score was defined as the sum of the scores of three paws (excluding the boosted paw); scores ranged from 0 to 12. (**B**) Tarsal joint tissues of CIA mice treated with fraxinellone or the control vehicle were stained with haematoxylin & eosin. The scale bars at the bottom right of the images indicate 200 μm. (**C**) Serum levels of IgG1 and IgG2a of CIA mice were determined using an enzyme-linked immunosorbent assay (ELISA). (**D**) Splenocytes of CIA mice were stimulated with or without anti-CD3 antibodies. The level of TNF-α and IFN-γ in culture media were measured by ELISA. Data are expressed as mean ± standard error of the mean (SEM). IgG1, immunoglobulin G1; IgG2a, immunoglobulin G2a; Frx, fraxinellone; TNF-α, tumor necrosis factor-α; IFN-γ, interferon-γ; Nil, no treatment; aCD3, stimulation with anti-CD3 antibodies; NS, not significant; ** *p* < 0.01; *** *p* < 0.001.

Either fraxinellone (7.5 mg/kg) or a vehicle control was administered intraperitoneally into CIA mice in order to evaluate the therapeutic effects of fraxinellone on inflammatory arthritis. The arthritis score was not significantly different between fraxinellone-treated mice and control CIA mice until five weeks after primary immunization. After five weeks, fraxinellone-treated CIA mice showed a mild form of inflammatory arthritis when compared to control CIA mice (Figure 1A). The difference in arthritis severity between CIA mice treated with fraxinellone or the vehicle control was maintained during the evaluation period. The tarsal joints of control CIA mice showed destruction of articular structures and inflammatory cell infiltration, whereas the joints of fraxinellone-treated CIA mice showed retained structure (Figure 1B). The serum level of immunoglobulin G (IgG) in fraxinellone-treated mice was also decreased (Figure 1C). To evaluate the effect of fraxinellone on cytokine production, splenocytes of CIA mice treated with fraxinellone or the vehicle control were stimulated with anti-cluster of differentiation (CD) 3 antibodies. The levels of TNF-α and interferon-γ (IFN-γ) in the culture supernatant were lower in the fraxinellone-treated group as compared to the control group, although the difference was not statistically significant (Figure 1D).

### 2.2. Fraxinellone Suppresses a Th17 Cell-Related Pathway

**Figure 2:**
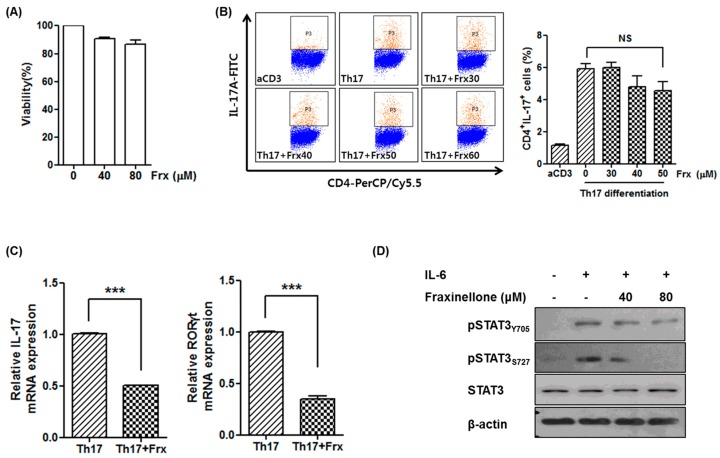
Fraxinellone inhibits Th17 differentiation. (**A**) Cell viability was evaluated using Cell Counting Kit-8 after treatment with 0, 40, and 80 μM of fraxinellone. Doses of fraxinellone with cell viability over 80% were considered tolerable. (**B**) CD4^+^ T cells were differentiated into Th17 cells in the presence of various doses of fraxinellone. The proportion of CD4^+^IL-17A^+^ cells was measured by flow cytometry. (**C**) Relative mRNA expression of *IL-17* and *RORγt* after treatment with or without fraxinellone was determined by reverse transcriptase-polymerase chain reaction (RT-PCR). CD4^+^ T cells were cultured with or without fraxinellone (40 μM) under Th17-favoring conditions. Data represent the mean of three independent experiments ± SEM. NS, not significant; *** *p* < 0.001. (**D**) Expression of STAT3 in CD4^+^ T cells in the presence or absence of fraxinellone was evaluated using Western blot analysis. CD4^+^ T cells were stimulated with IL-6, and the expression levels of STAT3 and phospho-STAT3 were determined by Western blot analysis. Frx, fraxinellone; aCD3, stimulation with anti-CD3 antibodies; RORγt, RAR-related orphan receptor γ t; STAT3, signal transducer and activator of transcription 3; pSTAT3_Y705_, phosphor-STAT3 at Tyrosine 705; pSTAT3_S727_, phosphor-STAT3 at Serine 727.

Since Th17 cells play a critical role in the pathogenesis of RA, we investigated the effect of fraxinellone on Th17 differentiation in vitro. To determine the dose of fraxinellone in a cell-based assay, an in vitro cytotoxicity assay was performed (Figure 2A). More than 80% of cells were viable until the concentration of fraxinellone was increased to 80 μM. Based on the cytotoxicity assay, CD4^+^ T cells isolated from murine spleens were cultured under Th17 differentiation conditions with a fraxinellone dose between 30 and 50 μM (Figure 2B). The proportion of CD4^+^IL-17^+^ cells was reduced by treatment with fraxinellone in a dose-dependent manner, however the difference was not statistically significant.

The expression of *IL-17* and *RAR-related orphan receptor gamma t* (*RORγt*) in CD4^+^ T cells was also evaluated in the absence or presence of 40 μM of fraxinellone. Although we did not observe a statistically significant inhibitory effect of fraxinellone on Th17 differentiation, the expression of *IL-17* and *RORγt* was markedly decreased by treatment with fraxinellone (Figure 2C). The expression of signal transducer and activator of transcription 3 (STAT3), one of the most important transcription factors in Th17 differentiation, was also investigated using Western blot analysis of T cells cultured with IL-6. The expression of phospho-STAT3 was suppressed in the presence of fraxinellone, suggesting that the signaling pathway associated with Th17 differentiation is downregulated by fraxinellone (Figure 2D).

### 2.3. Fraxinellone Controls B Cell Function

**Figure 3:**
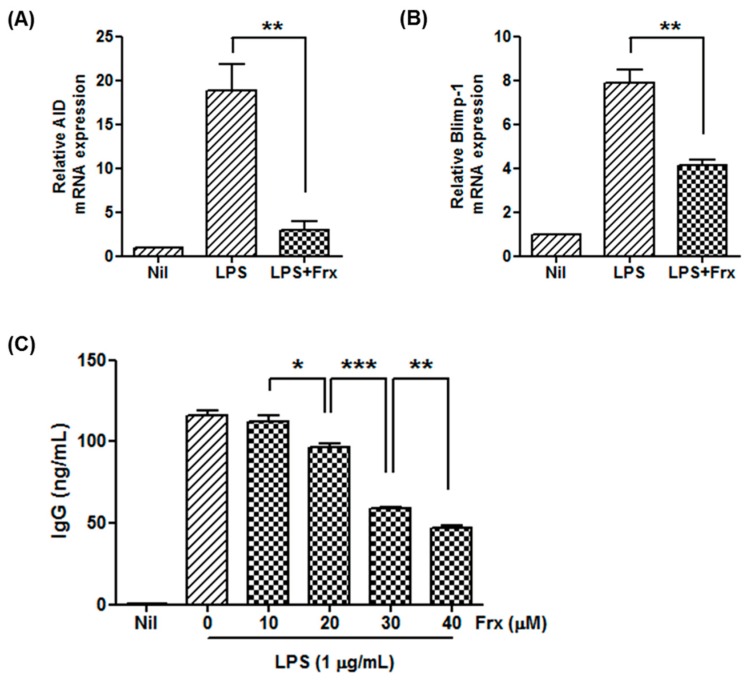
Fraxinellone inhibits B cell maturation. (**A**,**B**) CD19^+^ B cells were activated by lipopolysaccharide. After treatment with or without fraxinellone (40 μM), the relative expression levels of *AID* and *Blimp-1* in CD19^+^ B cells were determined with RT-PCR. (**C**) The level of immunoglobulin G in the culture supernatant was measured using ELISA in the presence of fraxinellone at doses of 0–40 μM. In all culture conditions, CD19^+^ B cells were stimulated by lipopolysaccharide. Data represent the mean of three independent experiments ± SEM. Nil, no stimulation; LPS, stimulation with lipopolysaccharide; Frx, fraxinellone; AID, activation-induced cytidine deaminase; Blimp-1, B lymphocyte-induced maturation protein-1; IgG, immunoglobulin G; * *p* < 0.05; ** *p* < 0.01; *** *p* < 0.001.

Because we observed reduced production of IgG in fraxinellone-treated CIA mice, we evaluated the effect of fraxinellone on immunoglobulin production and mRNA expression of CD19^+^ cells in vitro. The expression levels of *B lymphocyte-induced maturation protein-1* (*Blimp-1*) and *activation-induced cytidine deaminase* (*AID*), important transcription factors involved in development of germinal centers and antibody production, were upregulated after stimulation by lipopolysaccharides (LPS), but were significantly decreased by treatment with 40 μM of fraxinellone (Figure 3A,B). The production of immunoglobulin after LPS stimulation was also reduced by fraxinellone in a dose-dependent manner (Figure 3C).

### 2.4. Fraxinellone Inhibits Murine Osteoclastogenesis

CIA mice treated with fraxinellone showed a significant improvement in bone erosion as compared to control CIA mice. Because the production of IL-17, which is responsible for osteoclastogenesis, was inhibited by fraxinellone, we hypothesized that fraxinellone would inhibit osteoclastogenesis. To confirm the inhibitory effect of fraxinellone on osteoclastogenesis, murine bone marrow-derived monocytes were cultured with macrophage-colony stimulating factor (M-CSF) and RANKL, in the presence or absence of fraxinellone.

**Figure 4:**
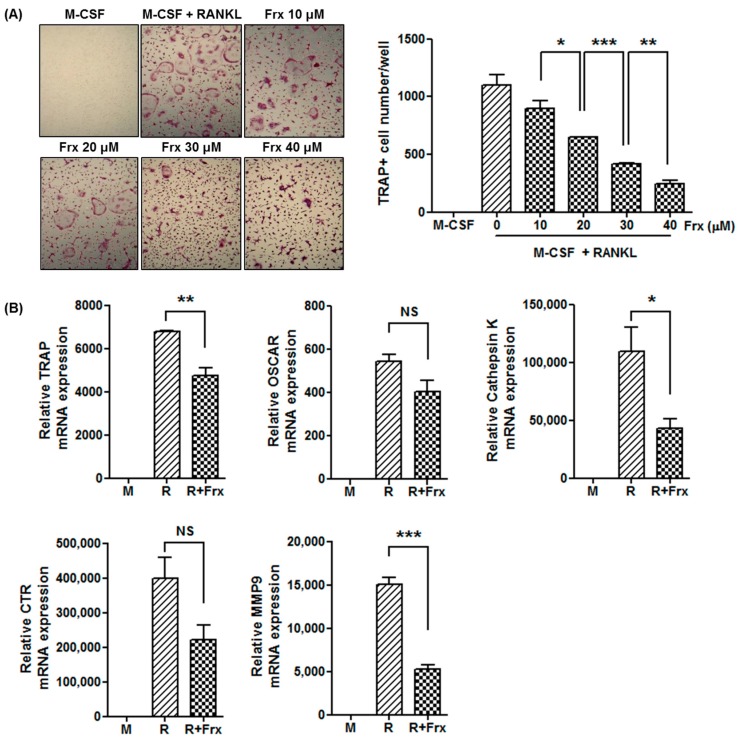
Fraxinellone suppresses murine osteoclastogenesis. (**A**) Murine monocytes obtained from the femur and tibia were cultured with M-CSF and RANKL to induce osteoclatogenesis. TRAP-positive multinucleated cells were counted in the culture dishes at various doses of fraxinellone (original magnification x100). (**B**) The relative mRNA levels of osteoclastogenesis-related markers, such as *TRAP*, *OSCAR*, *Cathepsin K*, *CTR*, and *MMP9*, were evaluated using RT-PCR. Data represent the mean of three independent experiments ± SEM. M-CSF, macrophage-colony stimulating factor; RANKL, receptor activator of nuclear factor-κB ligand; Frx, fraxinellone; TRAP, tartrate-resistant acid phosphatase; OSCAR, osteoclast-associated receptor; CTR, calcitonin receptor; MMP9, matrix metalloproteinase 9; M, culture with M-CSF; R, culture with M-CSF and RANKL; R + Frx, culture with M-CSF, RANKL, and fraxinellone; NS, not significant; * *p* < 0.05; ** *p* < 0.01; *** *p* < 0.001.

Monocyte cultures with various doses of fraxinellone between 10 to 40 μM showed a dose-dependent inhibition of osteoclast formation, which was determined by tartrate-resistant acid phosphatase (TRAP) staining (Figure 4A). The expression levels of osteoclastogenesis-related markers, such as *TRAP*, *cathepsin K*, and *matrix metalloproteinase 9* (*MMP9*), were significantly lower in the presence of 40 μM of fraxinellone (Figure 4B). The expression levels of other osteoclast markers, *osteoclast-associated immunoglobulin-like receptor* (*OSCAR*) and *calcitonin receptor*, were also reduced, although the difference was not statistically significant.

### 2.5. Fraxinellone Inhibits Th17 Differentiation and Osteoclastogenesis in Human

Based on the results from CIA mice, the inhibitory effects on Th17 differentiation and osteoclastogenesis were evaluated using peripheral blood mononuclear cells obtained from healthy controls.

**Figure 5:**
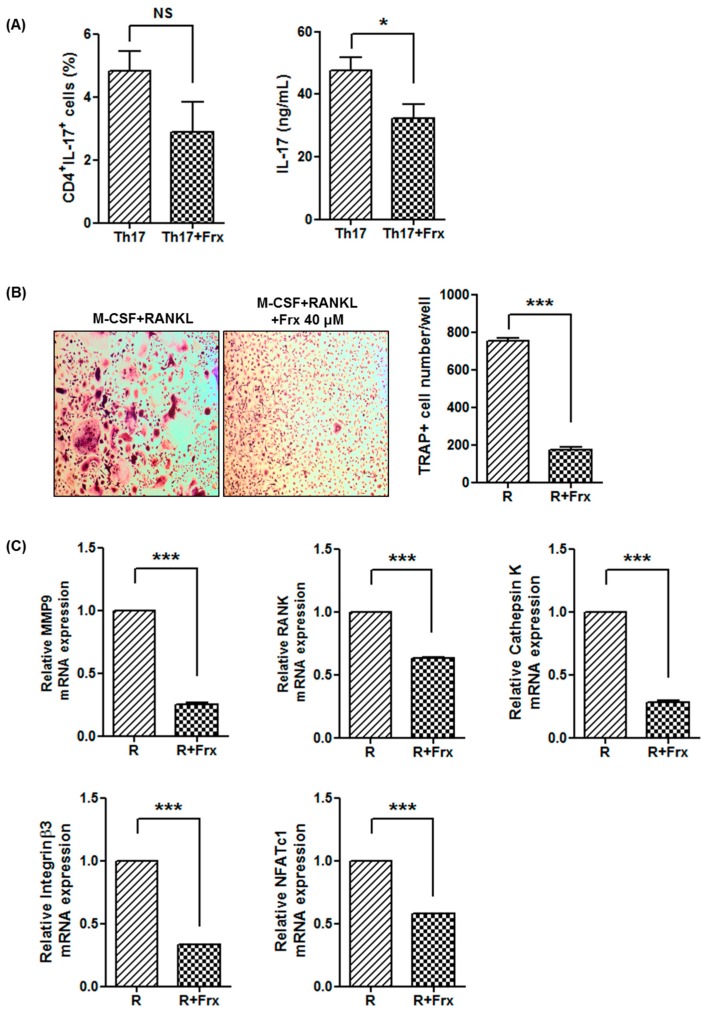
Fraxinellone regulates Th17 differentiation and osteoclastogenesis in human monocytes. (**A**) CD4^+^ T cells isolated from human peripheral blood mononuclear cells were cultured under Th17 differentiation conditions. After treatment with or without fraxinellone, the proportion of CD4^+^IL17^+^ T cells and IL-17 production were determined using flow cytometry and enzyme-linked immunosorbent assays, respectively. (**B**) Human peripheral blood mononuclear cells were cultured with M-CSF and RANKL to induce osteoclastogenesis. The number of TRAP-positive multinucleated cells was counted in the culture dishes with or without fraxinellone (40 μM) (original magnification ×100). (**C**) The expression levels of osteoclastogenesis-related markers, *MMP9*, *RANK*, *Cathepsin K*, *integrin β3*, and *NFATc1* were determined using RT-PCR in the absence or presence of fraxinellone (40 μM). Data represent the mean of three independent experiments ± SEM. Frx, fraxinellone; M-CSF, macrophage-colony stimulating factor; RANKL, receptor activator of nuclear factor-κB ligand; TRAP, tartrate-resistant acid phosphatase; MMP9, matrix metalloproteinase 9; RANK, receptor activator of nuclear factor-κB; NFATc1, nuclear factor of activated T-cells 1; R, culture with M-CSF and RANKL; R + Frx, culture with M-CSF, RANKL, and fraxinellone; NS, not significant; * *p* < 0.05; *** *p* < 0.001.

Human CD4^+^ T cells were cultured under Th17 differentiation condition with or without 40 μM of fraxinellone. Consistent with results from the animal experiments, the proportion of Th17 cells was not significantly different after treatment with fraxinellone, but the production of IL-17 was suppressed in the presence of fraxinellone (Figure 5A).

Fraxinellone also inhibited osteoclast differentiation from human peripheral blood mononuclear cells. Multinucleated cells stained with TRAP were significantly reduced in the presence of fraxinellone (Figure 5B). Fraxinellone also reduced the mRNA expression of osteoclastogenesis-related markers: *MMP9*, *RANK*, *cathepsin K*, *integrin β3*, and *nuclear factor of activated T-cells 1* (*NFATc1*) (Figure 5C). 

## 3. Discussion

Fraxinellone is a natural compound isolated from a widely distributed plant in Korea. In this study, fraxinellone showed a therapeutic effect on inflammatory arthritis. Fraxinellone alleviates synovial inflammation and osteoclast formation in CIA mice through inhibition of immune cells. This inhibitory effect was reproduced with in vitro experiments using human peripheral blood mononuclear cells. To the best of our knowledge, this is the first report that suggests an antiarthritic role of fraxinellone.

The root bark of *D. dasycarpus* has been a traditional herb used in the inflammatory conditions, such as colds, rheumatism, and jaundice. Recently, there have been efforts to delineate the underlying mechanisms of traditional medicines, and the anti-inflammatory effect of an active component of *D. dasycarpus*, fraxinellone, was evaluated in animal models with inflammatory diseases [12,13,15,16,17]. Previous research studies have proposed several mechanisms to explain the anti-inflammatory effects of fraxinellone.

In this study, we observed the inhibition of Th17 cell differentiation by fraxinellone. The expression levels of IL-17 and Th17 cell-related transcription factors were markedly reduced in the presence of fraxinellone. Although the proportion of Th17 cells showed no statistically significant difference between treatment with or without fraxinellone, there was a dose-dependent reduction. The critical transcription factors for Th17 differentiation, *RORγt* and phospho-STAT3, were downregulated by fraxinellone, which would explain inhibition of Th17 cells. A previous study had also suggested an inhibitory effect of fraxinellone on STAT1/3 expression in murine microglial cells [11]. Fraxinellone suppressed the activation of STAT1/3 signalling pathway triggered by viral components, leading to inhibition of inducible nitric oxide synthetase expression. Given that Th17 cells have a critical role in the pathogenesis of autoimmune diseases [2], fraxinellone may have a therapeutic effect in other inflammatory diseases.

However, inhibition of IL-17 does not guarantee the therapeutic effect in RA. Although the previous research provided a robust evidence for the therapeutic effect of inhibiting Th17 cell differentiation in animal studies, anti-IL-17 therapy failed to show the consistent therapeutic benefit in patients with active RA [18]. It is difficult to explain what makes this discrepancy. One study suggested that the highly variable expression of IL-17 in synovium between individual patients with RA could be responsible for inadequate response to anti-IL-17 therapy in subsets of patients [19]. Other possible explanations may be whether the drug targets Th17 cells or only IL-17. The previous research have suggested that IL-17 is not a potent inducer of inflammation by itself [20]. IL-17 accelerates the inflammatory response, in combination with other cytokines, such as TNF-α and IL-22 [21]. Th17 cells produce IL-22 as well as IL-17, and the synergistic activity of these cytokines might enhance the inflammatory reaction in RA. Given the importance of Th17 cells in the pathogenesis of RA, further investigations should be performed to elucidate the mechanisms underlying the inconsistent effect of anti-IL-17 therapy in RA patients.

Fraxinellone also inhibited the function of B cells in vivo and in vitro. Consistent with the lower serum levels of IgG in fraxinellone-treated CIA mice as compared to control CIA mice, in vitro experiments also showed fraxinellone-dependent suppression of IgG production from CD19^+^ B cells. The expression levels of critical transcription factors for B cell maturation were markedly downregulated in the presence of fraxinellone. AID and Blimp-1 play a key role for switch recombination/hypermutation in B cells and differentiation into plasma cells, respectively [22,23]. Thus, inhibition of *AID* and *Blimp-1* in B cells would reduce the production of autoantibodies in immune-mediated diseases. To our knowledge, there have been no data on the suppression of B cells by fraxinellone. Considering that B cell-depleting therapy is effective for the treatment of RA, the inhibition of B-cell function would be a credible mechanism for the therapeutic effect of fraxinellone on inflammatory arthritis. 

Previous studies have suggested that there is a fraxinellone-dependent inhibitory effect on macrophages under inflammatory conditions. Fraxinellone significantly reduced the expression of inducible nitric oxide synthetase and cyclooxygenase-2 through inhibition of nuclear factor-κB (NF-κB) in LPS-treated RAW264.7 cells. THP-1 cells and mouse primary peritoneal cells also showed downregulated expression of NF-κB signalling after treatment with fraxinellone [13]. NF-κB is a critical transcription factor that induces osteoclastogenesis [24,25]. Consistent with those previous observations, we observed the inhibition of osteoclastogenesis in the presence of fraxinellone. Because the therapeutic goal in RA treatment is to prevent joint destruction and to preserve joint function, the anti-osteoclastogenic effect of fraxinellone would provide better clinical outcomes in RA treatment.

Interestingly, fraxinellone seems to have an anti-inflammatory effect through inhibition of cellular differentiation or activation. In an animal model with T-cell-dependent hepatitis, fraxinellone induced apoptosis of activated CD4^+^ T cells in vivo and in vitro and inhibited the activation and infiltration of CD4^+^ T cells to the liver [15]. In a murine allergy model, the extract of *D. dasycarpus* significantly inhibited histamine release from activated mast cells [16]. Fraxinellone also controlled the activation of macrophages in mice with experimental colitis. The production of inflammatory mediators including IL-1β and nitric oxide from macrophages was significantly reduced by fraxinellone [13]. It is unclear whether the inhibitory effect of fraxinellone is limited to proinflammatory immune cells, or fraxinellone globally suppresses cellular differentiation and activation. Because drug safety is one of the most important issues for drug development, further investigation would be required to clarify the side effects of fraxinellone on normal cells.

In conclusion, fraxinellone has an inhibitory effect on synovial inflammation and osteoclastogenesis in mice with inflammatory arthritis. Fraxinellone could have a therapeutic effect through inhibition of cellular differentiation and activation. Although the safety profile should be determined, fraxinellone could be a novel treatment in inflammatory arthritis, such as RA.

## 4. Materials and Methods

### 4.1. Induction of CIA and Treatment with Fraxinellone

Male DBA/1J mice at 7 weeks of age (purchased from Charles River Laboratories, Yokohama, Japan) were injected intradermally at the base of the tail with 100 μg of type II collagen (CII) in complete Freund’s adjuvant (Chondrex, Redmond, WA, USA). Two weeks later, the mice were boosted in one hind footpad with 100 μg of CII emulsified in incomplete Freund’s adjuvant (Chondrex). The mice received intraperitoneal injection with either fraxinellone (7.5 mg/kg) or vehicle control (saline) three times per week beginning on day 14 after primary immunization. All procedures involving animals were in accordance with the Laboratory Animals Welfare Act, the Guide for the Care and Use of Laboratory Animals, and the Guidelines and Policies for Rodent Experimentation provided by the Institutional Animal Care and Use Committee of the Catholic University of Korea School of Medicine (CUMC-2014-0074-02, approved on 15 May 2014).

### 4.2. Assessment of Arthritis

The severity of inflammation was determined on a graded scale of 0 to 4 by three independent investigators as described previously [26]: 0 = no evidence of erythema and swelling; 1 = erythema and mild swelling confined to the mid-foot (tarsal) or ankle joint; 2 = erythema and mild swelling extending from the ankle to the mid-foot; 3 = erythema and moderate swelling extending from the ankle to the metatarsal joints; 4 = erythema and severe swelling encompassing the ankle, foot, and digits. The arthritic score was expressed as the sum of the scores from three limbs, excluding the boosted paw. Thus, the highest possible score was 12 points. Clinical arthritis was evaluated three times per week for eight weeks after primary immunization in order to investigate the effects of fraxinellone on CIA.

### 4.3. Histological Evaluation

Joint tissues of CIA mice were fixed in 10% paraformaldehyde, decalcified in Calci-Clear Rapid bone decalcifier (National Diagnostics, Atlanta, GA, USA), and embedded in paraffin. Tissue sections with 7-μm thickness were prepared and stained with hematoxylin and eosin (H&E). 

### 4.4. Analysis of Immunoglobulin G

Blood drawn from the orbital sinuses of mice was stored at −20 °C until use. The serum samples were diluted to 1:100,000 or 1:50,000 in Tris-buffered saline (pH 8.0) containing 1% bovine serum albumin and 0.5% Tween-20 for measurement of IgG1 or IgG2a, respectively. The concentrations of total IgG1 and IgG2a were measured using mouse IgG1 and IgG2a enzyme-linked immunosorbent assay (ELISA) Quantitation Kits (Bethyl Laboratories, Montgomery, TX, USA), respectively. Absorbance values were determined with an ELISA microplate reader operating at 450 nm.

### 4.5. Cytokine Measurement

Total splenocytes (1 × 10^6^ cells/mL/well) were stimulated with plate-bound anti-CD3 monoclonal antibodies (0.5 μg/mL) for 3 days. The levels of TNF-α, IFN-γ and IL-17 were determined using the DuoSet ELISA Development System (R&D Systems, Minneapolis, MN, USA).

### 4.6. Cytotoxicity

Total splenocytes (2 × 10^5^ cells/well) were seeded into 96-well flat-bottomed plates and stimulated with various doses (30, 40, 50 and 80 μM) of fraxinellone for 24 h. Two hours before termination of the culture, Cell Counting Kit-8 (Dojindo Molecular Technologies, Rockville, MD, USA) solution was added to the culture in order to evaluate the absorbance at 450 nm via a microplate reader.

### 4.7. Culture of CD4^+^ T Cells and CD19^+^ B Cells

Murine CD4^+^ T cells and CD19^+^ B cells were purified from spleens, and human CD4^+^ T cells were isolated from peripheral blood using a MACS isolation kit (Miltenyi Biotec Inc., Bergisch Gladbach, Germany) according to the manufacturer’s instructions. Protocols involving human samples were approved by the Institutional Review Board of Seoul St. Mary’s Hospital (KC15TISI0059, Approved on 15 March 2016). Cells were cultured in RPMI1640 media (Gibco, Grand Island, NY, USA) containing 10% fetal bovine serum (FBS).

Human or murine CD4^+^ T cells were treated with plate-bound anti-CD3 (0.5 μg/mL) and anti-CD28 (1 μg/mL) (BD PharMingen, San Diego, CA, USA). To evaluate the effect of fraxinellone on Th17 differentiation, CD4^+^ T cells were cultured with recombinant transforming growth factor-β (2 ng/mL) (PeproTech, Rocky Hill, NJ, USA), IL-6 (20 ng/mL), anti-interferon-γ (2 μg/mL), and anti-IL-4 (2 μg/mL) (R&D Systems) in the absence or presence of fraxinellone. Three days later, RNA was extracted for evaluation of mRNA expression, and culture media was obtained for measurement of IL-17.

The effect of fraxinellone on B cells was investigated with CD19^+^ B cells stimulated with 1 μg/mL LPS (Sigma-Aldrich, St. Louis, MO, USA) in the presence of fraxinellone. RNA and culture media were obtained for analysis after a 4-day culture.

### 4.8. Flow Cytometry

For intracellular detection of IL-17, CD4^+^ T cells were incubated with 25 ng/mL phorbol 12-myristate 13-acetate (PMA), 250 ng/mL ionomycin (Sigma-Aldrich), and monensin-containing GolgiStop (BD Biosciences, San Jose, CA, USA) for 4 h. The harvested cells were stained with PerCP-conjugated anti-CD4 antibodies (Biolegend, San Diego, CA, USA). After fixation with fixation/permeabilization solution, the cells were stained with 0.125 μg FITC-conjugated anti-IL-17 antibodies (eBioscience, San Diego, CA, USA) to determine the population of Th17 cells. All analyses were performed using a BD LSRII fortessa (BD Biosciences) and FACS DIVA version 10.0 (BD Biosciences). 

### 4.9. Real-Time Reverse Transcription Polymerase Chain Reaction (RT-PCR)

Total RNA was extracted using Trizol (Invitrogen, Carlsbad, CA, USA). PCR amplification and analyses were performed with a LightCycler 480 II instrument (Roche Life Science, Penzberg, Germany) according to the manufacturer’s instructions. LightCycler 480 SYBR Green I Master Mix (Roche Life Science) was used to develop all reactions. The primers for RT-PCR are given in Table 1. All mRNA expression levels were normalized to β-actin mRNA levels.

### 4.10. Western Blot Analysis

Murine splenocytes were treated with IL-6 (20 ng/mL), with or without fraxinellone for 30 min. At the given time, total cellular proteins from cells were extracted using RIPA buffer containing Halt Protease and Phosphatase Inhibitor Cocktail (Thermo scientific, Waltham, MA, USA). Polyacrylamide gel electrophoresis was performed at 100 V for 1.5 h, and proteins were transferred to polyvynilidene fluoride membrane (Bio-Rad, Hercules, CA, USA). To evaluate protein expression, membranes were incubated with the following antibodies: anti-STAT3, anti-phospho-STAT3_Y705_ (pSTAT3_Y705_), anti-pSTAT3_S727_ (Cell Signaling Technology, Danvers, MA, USA), and anti-β-actin antibodies (Sigma-Aldrich). Subsequently, the membranes were incubated with horseradish peroxidase-conjugated goat anti-rabbit IgG (Thermo Scientific) or goat anti-mouse IgG (Santa Cruz Biotechnology, Dallas, TX, USA). Reactive signals were evaluated using SuperSignal^®^ West Pico Chemiluminescent substrate (Thermo Scientific), and the membranes were then exposed to an Amersham Imager 600 (GE Healthcare Bioscience, Pittsburgh, PA, USA).

### 4.11. Osteoclastogenesis Assay

Bone marrow cells from mouse femurs and tibias and mononuclear cells isolated from peripheral blood of healthy humans were cultured in α-minimal essential medium (α-MEM; Invitrogen) containing antibiotics and 10% heat-inactivated FBS. To induce osteoclastogenesis, the floating murine monocytes and adherent human monocytes were harvested and cultured with 100 ng/mL recombinant M-CSF (R&D Systems) for 3 days. The monocytes were further stimulated with 25 ng/mL of M-CSF and 50 ng/mL of RANKL in the absence or presence of fraxinellone. On day 2, the culture media was replaced with fresh medium containing the same components. After a 4-day culture, monocytes were harvested for isolation of RNA or fixation with paraformaldehyde. The fixed cells were stained with TRAP using a commercial kit (Sigma-Aldrich) according to the manufacturer’s instructions, omitting the counterstaining with hematoxylin. TRAP-positive cells containing three or more nuclei were counted under a light microscope.

### 4.12. Statistical Analysis

The experimental data are presented as the mean and standard error. Statistical significance was determined using the Student’s *t*-test, and *p*-values < 0.05 were considered significant. All data were analyzed using SAS software (v. 9.1; SAS Institute, Cary, NC, USA) and GraphPad Prism software (v. 5.01; GraphPad, San Diego, CA, USA).

## Figures and Tables

**Table 1:** Primer sequences for real-time RT-PCR.

Gene		Sense (5′–3′)	Anti-Sense (5′–3′)
Mouse	*IL-17*	CCTCAAAGCTCAGCGTGTCC	GAGCTCACTTTTGCGCCAAG
	*RORγt*	TGTCCTGGGCTACCCTACTG	GTGCAGGAGTAGGCCACATT
	*Blimp-1*	CTGTCAGAACGGGATGAACA	TGGGGACACTCTTTGGGTAG
	*AID*	CGTGGTGAAGAGGAGAGATAGTG	CAGTCTGAGATGTAGCGTAGGAA
	*TRAP*	TCCTGGCTCAAAAAGCAGTT	ACATAGCCCACACCGTTCTC
	*OSCAR*	CCTAGCCTCATACCCCCAG	CAAACCGCCAGGCAGATTG
	*Cathepsin K*	CAGCAGAGGTGTGTACTATG	GCGTTGTTCTTACTTCGAGC
	*CTR*	CGGACTTTGACACAGCAGAA	AGCAGCAATCGACAAGGAGT
	*MMP9*	CTGTCCAGACCAAGGGTACAGCCT	GAGGTATAGTGGGACACATAGTGG
	*β-Actin*	GAAATCGTGCGTGACATCAAAG	TGTAGTTTCATGGATGCCACAG
Human	*MMP9*	TGGGGGGCAACTCGGC	GGAATGATCTAAGCCCAG
	*Cathepsin K*	TGAGGCTTCTCTTGGTGTCCATAC	AAAGGGTGTCATTACTGCGGG
	*Integrin β3*	GCAATGGGACCTTTGAGTGT	GTGGCAGACACATTGACCAC
	*RANK*	GCTCTAACAAATGTGAACCA	GCCTTGCCTGTATCACAAAC
	*NFATc1*	GCATCACAGGGAAGACCGTGTC	GAAGTTCAATGTCGGAGTTTCTGAG
	*β-actin*	GGACTTCGAGCAAGAGATGG	TGTGTTGGGGTACAGGTCTTTG

IL-17, interleukin-17; RORγt, RAR-related orphan receptor γ t; Blimp-1, B lymphocyte-induced maturation protein-1; AID, activation-induced cytidine deaminase; TRAP, tartrate resistant acid phosphatase; OSCAR, osteoclast-associated immunoglobulin-like receptor; CTR, calcitonin receptor; MMP9, matrix metalloproteinase 9; RANK, receptor activator of nuclear factor-κB; NFATc1, nuclear factor of activated T-cells 1.

## Abbreviations

RA	Rhematoid arthritis
IL	Interleukin
Th17	T helper cell producing IL-17
TNF-α	Tumor necrosis factor-alpha
RANK	Receptor activator of nuclear factor-κB
RANKL	Receptor activator of nuclear factor-κB ligand
DMARD	Disease modifying anti-rheumatic drug
CIA	Collagen-induced arthritis
Ig	Immunoglobulin
IFN-γ	Interferon-gamma
ELISA	Enzyme-linked immunosorbent assay
SEM	Standard error of the mean
RORγt	RAR-related orphan receptor γ t
STAT3	Signal transducer and activator of transcription 3
pSTAT3	Phospho-signal transducer and activator of transcription 3
RT-PCR	Reverse transcriptase-polymerase chain reaction
Blimp-1	B lymphocyte-induced maturation protein-1
AID	Activation-induced cytidine deaminase
LPS	Lipopolysaccharide
M-CSF	Macrophage-colony stimulating factor
TRAP	Tartrate resistant acid phosphatase
MMP9	Matrix metalloproteinase 9
OSCAR	Osteoclast-associated immunoglobulin-like receptor
CTR	Calcitonin receptor
NFATc1	Nuclear factor of activated T-cells 1.
NF-κB	Nuclear factor-κB

## References

[1] McInnes I.B., Schett G. (2011). The pathogenesis of rheumatoid arthritis. N. Engl. J. Med..

[2] Miossec P., Korn T., Kuchroo V.K. (2009). Interleukin-17 and type 17 helper T cells. N. Engl. J. Med..

[3] Cohen S.B., Emery P., Greenwald M.W., Dougados M., Furie R.A., Genovese M.C., Keystone E.C., Loveless J.E., Burmester G.R., Cravets M.W. (2006). Rituximab for rheumatoid arthritis refractory to anti-tumor necrosis factor therapy: Results of a multicenter, randomized, double-blind, placebo-controlled, phase III trial evaluating primary efficacy and safety at twenty-four weeks. Arthritis Rheum..

[4] Emery P., Fleischmann R., Filipowicz-Sosnowska A., Schechtman J., Szczepanski L., Kavanaugh A., Racewicz A.J., van Vollenhoven R.F., Li N.F., Agarwal S. (2006). The efficacy and safety of rituximab in patients with active rheumatoid arthritis despite methotrexate treatment: Results of a phase IIB randomized, double-blind, placebo-controlled, dose-ranging trial. Arthritis Rheum..

[5] Scott D.L., Wolfe F., Huizinga T.W. (2010). Rheumatoid arthritis. Lancet.

[6] Jiang Y., Li S.P., Chang H.T., Wang Y.T., Tu P.F. (2006). Pressurized liquid extraction followed by high-performance liquid chromatography for determination of seven active compounds in Cortex Dictamni. J. Chromatogr. A.

[7] Sun J., Wang X., Wang P., Li L., Qu W., Liang J. (2015). Antimicrobial, antioxidant and cytotoxic properties of essential oil from *Dictamnus angustifolius*. J. Ethnopharmacol..

[8] Jeong G.S., Byun E., Li B., Lee D.S., Kim Y.C., An R.B. (2010). Neuroprotective effects of constituents of the root bark of *Dictamnus dasycarpus* in mouse hippocampal cells. Arch. Pharm. Res..

[9] Yoon J.S., Yang H., Kim S.H., Sung S.H., Kim Y.C. (2010). Limonoids from *Dictamnus dasycarpus* protect against glutamate-induced toxicity in primary cultured rat cortical cells. J. Mol. Neurosci..

[10] Kim J.H., Park Y.M., Shin J.S., Park S.J., Choi J.H., Jung H.J., Park H.J., Lee K.T. (2009). Fraxinellone inhibits lipopolysaccharide-induced inducible nitric oxide synthase and cyclooxygenase-2 expression by negatively regulating nuclear factor-κB in RAW 264.7 macrophages cells. Biol. Pharm. Bull..

[11] Lee C.S., Won C., Yoo H., Yi E.H., Cho Y., Maeng J.W., Sung S.H., Ye S.K., Chung M.H. (2009). Inhibition of double-stranded RNA-induced inducible nitric oxide synthase expression by fraxinellone and sauchinone in murine microglia. Biol. Pharm. Bull..

[12] Kim H., Kim M., Kim H., Lee G.S., An W.G., Cho S.I. (2013). Anti-inflammatory activities of *Dictamnus dasycarpus* Turcz., root bark on allergic contact dermatitis induced by dinitrofluorobenzene in mice. J. Ethnopharmacol..

[13] Wu X.F., Ouyang Z.J., Feng L.L., Chen G., Guo W.J., Shen Y., Wu X.D., Sun Y., Xu Q. (2014). Suppression of NF-κB signaling and NLRP3 inflammasome activation in macrophages is responsible for the amelioration of experimental murine colitis by the natural compound fraxinellone. Toxicol. Appl. Pharmacol..

[14] Han X., Chen H., Zhou J., Tai H., Gong H., Wang X., Huang N., Qin J., Fang T., Wang F. (2017). The inhibitory effect in Fraxinellone on oxidative stress-induced senescence correlates with AMP-activated protein kinase-dependent autophagy restoration. J. Cell. Physiol..

[15] Sun Y., Qin Y., Gong F.Y., Wu X.F., Hua Z.C., Chen T., Xu Q. (2009). Selective triggering of apoptosis of concanavalin A-activated T cells by fraxinellone for the treatment of T-cell-dependent hepatitis in mice. Biochem. Pharmacol..

[16] Jiang S., Nakano Y., Rahman M.A., Yatsuzuka R., Kamei C. (2008). Effects of a *Dictamnus dasycarpus* T. extract on allergic models in mice. Biosci. Biotechnol. Biochem..

[17] Yang B., Lee H.B., Kim S., Park Y.C., Kim K., Kim H. (2017). Decoction of *Dictamnus Dasycarpus* Turcz. Root Bark Ameliorates Skin Lesions and Inhibits Inflammatory Reactions in Mice with Contact Dermatitis. Pharmacogn. Mag..

[18] Kunwar S., Dahal K., Sharma S. (2016). Anti-IL-17 therapy in treatment of rheumatoid arthritis: A systematic literature review and meta-analysis of randomized controlled trials. Rheumatol. Int..

[19] Van Baarsen L.G., Lebre M.C., van der Coelen D., Aarrass S., Tang M.W., Ramwadhdoebe T.H., Gerlag D.M., Tak P.P. (2014). Heterogeneous expression pattern of interleukin 17A (IL-17A), IL-17F and their receptors in synovium of rheumatoid arthritis, psoriatic arthritis and osteoarthritis: Possible explanation for nonresponse to anti-IL-17 therapy?. Arthritis Res. Ther..

[20] Gaffen S.L. (2009). Structure and signalling in the IL-17 receptor family. Nat. Rev. Immunol..

[21] Veldhoen M. (2017). Interleukin 17 is a chief orchestrator of immunity. Nat. Immunol..

[22] Muramatsu M., Kinoshita K., Fagarasan S., Yamada S., Shinkai Y., Honjo T. (2000). Class switch recombination and hypermutation require activation-induced cytidine deaminase (AID), a potential RNA editing enzyme. Cell.

[23] Turner C.A., Mack D.H., Davis M.M. (1994). Blimp-1, a novel zinc finger-containing protein that can drive the maturation of B lymphocytes into immunoglobulin-secreting cells. Cell.

[24] Lee Z.H., Kim H.H. (2003). Signal transduction by receptor activator of nuclear factor κB in osteoclasts. Biochem. Biophys. Res. Commun..

[25] Jimi E., Ghosh S. (2005). Role of nuclear factor-κB in the immune system and bone. Immunol. Rev..

[26] Brand D.D., Latham K.A., Rosloniec E.F. (2007). Collagen-induced arthritis. Nat. Protoc..

